# Leaffooted Bugs, *Leptoglossus phyllopus* (Hemiptera: Coreidae), Are Attracted to Volatile Emissions from Herbivore-Damaged Cotton Bolls

**DOI:** 10.3390/insects16040425

**Published:** 2025-04-17

**Authors:** Malek A. Alwedyan, Anjel M. Helms, Michael J. Brewer

**Affiliations:** 1 Department of Entomology, Texas A&M AgriLife Research and Extension Center, Corpus Christi, TX 78406, USA; malek.alwedyan@ag.tamu.edu; 2 Department of Entomology, Texas A&M University, College Station, TX 77843, USA; anjel.helms@ag.tamu.edu

**Keywords:** behavioral responses, herbivore-induced plant volatiles, host attraction, semiochemicals, volatile organic compounds

## Abstract

The leaffooted bug, *Leptoglossus phyllopus* (L.) (Hemiptera: Coreidae), probes and feeds on tissues of many plant species, including developing cotton bolls, causing seed damage and abscission. Insecticides are the primary tool for managing leaffooted bugs, but concerns about resistance and environmental harm highlight the need for alternative management strategies. One promising approach is using semiochemicals, such as plant- and insect-produced volatile organic compounds (VOCs), to trap or repel pests. Insect herbivores often use plant-produced VOCs to select suitable host plants for feeding and oviposition. Field observations of abundant adult leaffooted bugs on cotton bolls suggest that bugs aggregate at feeding sites. The goal of this study was to characterize VOCs from developing cotton bolls with and without leaffooted bug herbivory and evaluate how these VOCs affect adult bug foraging behavior. A portable dynamic headspace sampling method was used to collect VOCs from developing cotton bolls in the field, and VOC samples were analyzed using gas chromatography–mass spectrometry. Leaffooted bug herbivory induced volatile emissions from cotton bolls, with significant increases in the emissions of six compounds (benzaldehyde, α-pinene, β-pinene, β-myrcene, p-xylene, and (*E*)-β-caryophyllene). Dual-choice olfactometer assays revealed that adult leaffooted bugs were attracted to VOCs from damaged cotton bolls, as well as being attracted to synthetic benzaldehyde or α-pinene individually. In contrast, leaffooted bugs were repelled by the combination of synthetic benzaldehyde and α-pinene. These findings suggest that VOCs from cotton bolls are attractive to leaffooted bugs and could contribute to the development of attractive lures for integrated pest management.

## Simple Summary

Insect herbivores often use plant-produced VOCs to select suitable host plants for feeding and oviposition. The leaffooted bug probes and feeds on tissues of many plant species, including developing cotton bolls, causing seed damage and boll abscission. Field observations of abundant adult leaffooted bugs on cotton bolls suggest that bugs aggregate at feeding sites. The goal of this study was to characterize VOCs from leaffooted bug-damaged cotton bolls and evaluate how these VOCs affect adult bug foraging behavior. A portable dynamic headspace sampling method was used to collect VOCs from developing cotton bolls in the field and VOC samples were analyzed using gas chromatography-mass spectrometry. Leaffooted bug herbivory induced volatile emissions from cotton bolls, with significant increases in the emissions of six compounds (benzaldehyde, α-pinene, β-pinene, β-myrcene, p-xylene, and (*E*)-β-caryophyllene). Dual-choice olfactometer assays revealed that adult leaffooted bugs were attracted to herbivore-induced VOCs, as well as being attracted to synthetic benzaldehyde or α-pinene individually. These findings suggest that selected cotton VOCs could contribute to the development of attractive lures.

## 1. Introduction

The leaffooted bug, *Leptoglossus phyllopus* (L.) (Hemiptera: Coreidae), is among the five most extensively studied species in the *Leptoglossus* genus [1]. This polyphagous pest has piercing–sucking mouthparts that inject proteinaceous saliva into developing seeds and fruits, causing significant injury [1]. Both adults and nymphs can directly damage marketable fruiting bodies through feeding, leading to seed injury, fruit drop, discoloration, and necrosis [2]. In addition to causing direct damage, leaffooted bugs can transmit plant diseases and make plants more susceptible to secondary infections [2]. Moreover, leaffooted bug species have multiple scent glands capable of releasing a variety of semiochemical blends, which are commonly associated with plant defenses. These semiochemical blends influence a wide range of behaviors, including feeding, aggregation, and mating [1].

Over the past two decades, damage to cotton bolls caused by herbivore feeding has increased significantly in the southern United States, including Texas [3]. Leaffooted bugs, including *L. phyllopus*, and several stink bug species (Hemiptera: Pentatomidae) are key members of the boll-feeding bug complex, infesting cotton at high levels across the Cotton Belt and causing damage to bolls throughout much of the season [4]. Cotton, *Gossypium hirsutum* (L.) (Malvales: Malvaceae), encounters significant pressure from pest insects, requiring extensive use of synthetic pesticides [5]. Insecticides are the primary method for controlling bugs in the cotton boll–feeding pest complex, often requiring two or more applications each season [4]. There is a growing demand for alternative pest control strategies to enhance the economic and environmental sustainability of agricultural systems [6].

Semiochemical-based pest management offers a promising, more environmentally friendly alternative to broad-spectrum insecticide use [7]. Unlike chemical insecticides, volatile semiochemicals that exploit pest behavioral responses pose a lower risk of resistance development in insects [8]. Semiochemicals from several leaffooted bug species have been characterized, including aggregation pheromones from *L. phyllopus.* However, previous efforts to develop attractants for baited trapping have been inconclusive [1]. For example, lures combining both pheromones and host-plant volatiles have been found to successfully attract some pest species, such as *Harlequin bug*, *Murgantia histrionica* (Hahn) (Hemiptera: Pentatomidae) [9]. This approach of incorporating plant volatiles with insect semiochemicals may also be effective for *L. phyllopus*.

Plants respond to environmental cues, including insect herbivore attacks, by synthesizing and releasing volatile organic compounds (VOCs) into the atmosphere [10]. Herbivores often rely on plant VOCs, directed by their presence and relative proportions [11] for essential activities such as locating food, seeking shelter, or oviposition [12]. When plants are attacked by herbivores, they may release VOCs in response to herbivory, referred to as herbivore-induced plant volatiles (HIPVs). Depending on ecological and physiological context, HIPVs can act as either attractants or repellents for foraging herbivores [13,14]. These HIPVs may indicate the suitability of that plant for other herbivores [15]. Conversely, some foraging herbivores avoid HIPVs to evade competition, escape natural enemies, or avoid plants with poor nutritional quality due to induced defenses [16]. Recent studies have characterized HIPVs from leaffooted bug herbivory on cotton [17], but little is known about influence of these compounds on leaffooted bug behavior.

The chemical composition of cotton plants has been extensively studied because of their economic importance and susceptibility to attack from various herbivores [18]. Cotton plants have served as a model for studying the dynamics and functions of VOC emissions [19]. Herbivore-damaged cotton plants release HIPVs primarily from the site of damage, which has been observed in both leaves and reproductive structures such as cotton bolls. In this study, we focused on cotton bolls due to their economic importance in relation to leaffooted bug herbivory [20,21]. For example, a laboratory study found that feeding by *Euschistus servus* (Say) and the southern green stink bug, *Nezara viridula* (Linnaeus) (Hemiptera: Pentatomidae), significantly increased volatile emissions from bolls compared to undamaged bolls. Damaged bolls released substantially higher amounts of acyclic terpenes and methyl ketones than undamaged bolls [22]. Furthermore, studies have highlighted the role of cotton volatile blends in influencing herbivore foraging behavior [23]. For instance, adult boll weevils, *Anthonomus grandis* (Boheman) (Coleoptera: Curculionidae), were attracted to HIPVs emitted by cotton plants damaged by conspecifics (individuals of the same species). This suggests that *A. grandis* uses these volatiles to locate hosts and may prefer them during its reproductive stage [24].

The research presented here aimed to characterize the VOCs released by developing cotton bolls in response to herbivory by adult leaffooted bugs under field conditions and to evaluate how these VOCs influence their foraging behavior. Developing cotton bolls are especially vulnerable to feeding by adult leaffooted bugs and serve as a preferred feeding site. Therefore, VOC collections were conducted at this stage, as it represents a key interval of susceptibility targeted by leaffooted bugs under field conditions. We hypothesized that the VOC blends emitted by developing cotton bolls would differ, with an increased emission of VOCs following herbivory by adult leaffooted bugs compared to non-damaged (control) cotton bolls. Since adult leaffooted bugs commonly feed in aggregations [1], we also expected that HIPV emissions from developing cotton bolls would attract conspecific adults. We collected VOCs from developing cotton bolls, either damaged by adult leaffooted bugs or left non-damaged (control), using a headspace sampling system adapted for field use. Subsequent analysis was performed using gas chromatography–mass spectrometry (GC-MS) to identify and quantify the compounds in the VOC blends from both herbivory treatments. The behavioral responses of adult leaffooted bugs to herbivore-induced cotton volatiles were assessed using a dual-choice Y-tube olfactometer. Understanding the role of VOCs in mediating the foraging behavior of adult leaffooted bugs could contribute to developing semiochemical-based management strategies.

## 2. Materials and Methods

### 2.1. Plants and Insects

In mid-April 2023, a commercial cotton cultivar, DeltaPine 2020 B3XF (Bayer Crop Science, St. Louis, MO, USA), was planted in Corpus Christi, Texas. This cultivar was selected because it is commercially grown in the southern USA, and leaffooted bugs have previously been observed feeding on it under field conditions. Planting was scheduled at the end of the local planting window to increase the likelihood of leaffooted bug presence. After emergence, cotton plants were thinned to maintain a spacing of 45.7 cm between plants within each of the 10 rows of the experimental plot. Each plot was 5 m long, with rows spaced 0.96 m apart. The entire experimental area was planted with the same cultivar and managed according to standard regional practices, except that no insecticides were applied.

Adult leaffooted bugs used in experiments were collected from field populations in a cotton nursery and placed in plastic portion cups. Leaffooted bugs were collected and used for infestation on the same day. Approximately 70 days after planting, cotton plants were caged using organza fabric cages (40 × 30 cm, ~240 μm mesh; JoAnn Fabrics, Hudson, OH, USA) to protect developing bolls from leaffooted bugs feeding. The organza fabric cages were removed at the initiation of collection experiments. Leaffooted bugs were starved for 2 h prior to the experiment. Cotton bolls were infested by placing adult leaffooted bugs for continuous feeding over a 24 h period inside fine mesh fabric cages (13 × 15 cm, ~0.2 mm mesh; Hobby Lobby Stores, Corpus Christi, TX, USA) placed over selected bolls and secured with twist ties. Plants in the damaged treatment received four adult leaffooted bugs (the sex of the leaffooted bugs was not determined). Non-damaged control bolls were enclosed in empty fine mesh fabric cages without leaffooted bugs, also for 24 h. After the feeding period, the cages and adult leaffooted bugs were carefully removed to avoid causing mechanical damage to the bolls. For each VOC collection, only one boll per plant was used.

### 2.2. Plant Volatile Organic Compound Collection

Volatile organic compounds were collected from developing cotton bolls that were either damaged by adult leaffooted bugs (*n* = 12) or remained undamaged as control bolls (*n* = 12). A portable dynamic headspace sampling device was used for VOC collection. Heat-resistant oven bags (30 × 20 cm; Reynolds Kitchens Turkey Oven Bags; Lake Forest, IL, USA) were placed over individual bolls and bag openings were secured with a pipe cleaner [25]. Air was introduced into each oven bag at a flow rate of 45 mL/min through an inlet volatile filter trap (VFT), which served to eliminate potential contaminants before air entered the oven bag, while simultaneously being pulled through an outlet VFT at a rate of 20 mL/min using a vacuum pump. Before initiating field VOC collections, we conducted preliminary laboratory tests to evaluate the efficiency of the VFTs and to determine the appropriate sampling duration for cotton bolls. These tests confirmed that the VFTs effectively captured the target VOCs without detectable breakthrough under our experimental conditions. Based on these results, the probability of breakthrough was considered minimal. Furthermore, the findings we present compare the relative emission rates from damaged and control bolls and not absolute quantifications. Volatiles were collected on a VFT containing 45 mg of HayeSep^®^ Q (Hutchison Hayes Separation Inc., Houston, TX, USA) during a 5 h period from 11:00 to 16:00. After collections, VFTs were removed and stored at room temperature in aluminum foil. The study also included a smaller number of control collections conducted in the laboratory. These included adult leaffooted bugs without developing cotton bolls (*n* = 3); the sex of the leaffooted bugs was not determined, and each replicate contained three adult bugs of mixed sex, as well as empty oven bags without developing cotton bolls or adult leaffooted bugs (*n* = 3). These control collections were used to check for non-plant VOC contaminants and to exclude such compounds from the dataset. During the VOC collection period, the average temperature was 30 °C, the wind speed was 16 mph, and the relative humidity was 80%.

### 2.3. Volatile Organic Compounds Analysis

Volatile organic compounds were analyzed using gas chromatography coupled with mass spectrometry (GC-MS). Volatile organic compounds were eluted from the outlet VFTs (located at the exit of the airflow system) using 150 µL of dichloromethane. To each sample, 5 µL of nonyl acetate (400 ng) was added as an internal standard. Quantification of VOCs was performed using an Agilent 7890B gas chromatograph coupled with a 5977B mass spectrometer (Agilent Technologies, Santa Clara, CA, USA). The analysis utilized a splitless injection with the inlet set at 250 °C, and helium was used as a carrier gas with a flow rate of 1.0 mL/min. After injecting a 1 µL sample, the column (HP-5MS 30 m × 0.250 mm ID, 0.25 µm film thickness; Agilent Technologies, Santa Clara, CA, USA) was held at 40 °C for 5 min. The temperature then increased by 20 °C per minute until reaching 250 °C. Compounds were ionized via electron impact at 70 eV, and mass spectra were collected by scanning from 40 to 300 m/z at a rate of 5.30 scans per second. Target VOCs were tentatively identified by comparing them with mass spectral libraries (NIST17 and Adams2), and structural assignments were confirmed by comparing the mass spectra and retention times with those of authentic standards. The relative abundance of each compound, measured in ng, was calculated using an internal standard [26].

### 2.4. Olfactometer Bioassays

A Y-tube olfactometer was utilized to evaluate the preference of adult leaffooted bugs for VOC emissions from cotton bolls. The olfactometer consisted of a transparent glass tube with a 3 cm diameter, comprising a 15 cm long central section and two lateral arms, each measuring 20 cm in length and angled at 45°. To provide consistent lighting and minimize visual distractions, the olfactometer was placed inside a cardboard box. The box had two openings: one at the top for upper lighting and one at the front for researcher access and to facilitate the use of the olfactometer. The olfactometer was positioned centrally within the box, ensuring no directional bias. A spotlight positioned outside the box delivered uniform, diffused illumination throughout the experiment. Air was transported to each arm of the olfactometer at a constant flow rate of 20 mL/min using vacuum pumps (AIRPO; SparkFun Electronics; Niwot, CO, USA) and monitored for accuracy with flowmeters. Each arm was connected to an oven bag containing the VOC source, with all connections created using Teflon tubing. An individual adult leaffooted bug (sex not determined) was introduced at the Y-tube entrance and monitored for 10 min to record its choice between the two arms. For those that reached the end of an arm, their choice was documented. Conversely, individuals that stayed near the entrance without moving for the entire 10 min duration were marked as showing ‘no response’ and excluded from the statistical analysis. Prior to the behavioral bioassay, adult leaffooted bugs were starved for 2 h. To ensure unbiased results, each adult leaffooted bug was tested only once. Random Forest (RF; see Section 2.5) analysis identified benzaldehyde and α-pinene as key compounds in the volatile blends emitted by cotton bolls in response to adult leaffooted bug herbivory, encouraging their selection for subsequent bioassays.

Five bioassays were conducted to assess the behavioral response of adult leaffooted bugs: Bioassay 1: non-damaged (control) bolls against air blank (*n* = 36); Bioassay 2: non-damaged (control) bolls against bolls damaged by adult leaffooted bug (*n* = 36); Bioassay 3: benzaldehyde against dichloromethane (*n* = 36); Bioassay 4: α-pinene against dichloromethane (*n* = 36); and Bioassay 5: benzaldehyde and α-pinene against dichloromethane (*n* = 36). Synthetic solutions of benzaldehyde and α-pinene were prepared to mimic the volatile concentrations emitted by cotton bolls damaged with leaffooted bugs during a 5 h collection period. For individual compound tests, 10 µL of each compound solution was applied to a filter paper, while control treatments received 10 µL of the solvent alone. For the combination treatment, 10 µL of each compound (20 µL total) was applied to the same filter paper, and the control received 20 µL of solvent. The synthetic compounds benzaldehyde (≥99.5%) and α-pinene (≥90%) were used, along with dichloromethane (≥99.8%) and Hexane (≥98.5%). All chemicals were sourced from Sigma-Aldrich (Milwaukee, WI, USA). To minimize positional bias, the positions of the VOC sources (cotton bolls, synthetic compounds, and control) in the Y-tube olfactometer were changed between the left and right arms after every sixth adult leaffooted bug was tested. This ensured that each VOC source was presented approximately equally on both sides. To prevent VOC contamination, the olfactometer was cleaned with hexane and air-dried for 15 min after every sixth adult leaffooted bug was tested. Fresh filter paper disks with newly prepared VOC treatments were also used after every sixth adult leaffooted bug to maintain consistency and to prevent bias.

### 2.5. Statistical Analyses

All analyses were performed using R Studio (R Version 3.6.1 [27]). A permutational analysis of variance (PERMANOVA) was performed using the vegan package (version 2.6-10) and the function adonis to quantify the differences in VOC blends between developing cotton bolls damaged by adult leaffooted bugs and non-damaged (control) developing cotton bolls [28]. Non-metric multidimensional scaling (NMDS) was conducted using the vegan package in R Studio to visualize variations in VOC blends [29]. Random Forest analysis was conducted using the randomForest package in R Studio to identify the compounds that distinguished the volatile blends between the herbivory treatments [30,31]. The VOC emission data from cotton bolls were first tested for normality using the Shapiro–Wilk test. As the data did not meet the assumption of normality, a log transformation was applied, which improved the distribution. Subsequent analyses were conducted using Student’s *t*-tests on the log-transformed data. These tests were used to assess statistically significant differences in total VOC emissions and individual compound emissions between developing cotton bolls damaged by adult leaffooted bugs and undamaged (control) bolls. Volatiles were included in analyses if they were found to occur in at least 50% of samples within a treatment. Chi-square goodness-of-fit analysis was conducted at a significance level of *p* < 0.05 to evaluate the Y-tube olfactometer assays.

## 3. Results

### 3.1. Adult Leaffooted Bug Herbivory Induced Quantitative Changes the Blend of VOCs Emitted from Cotton Bolls

**Figure 1:**
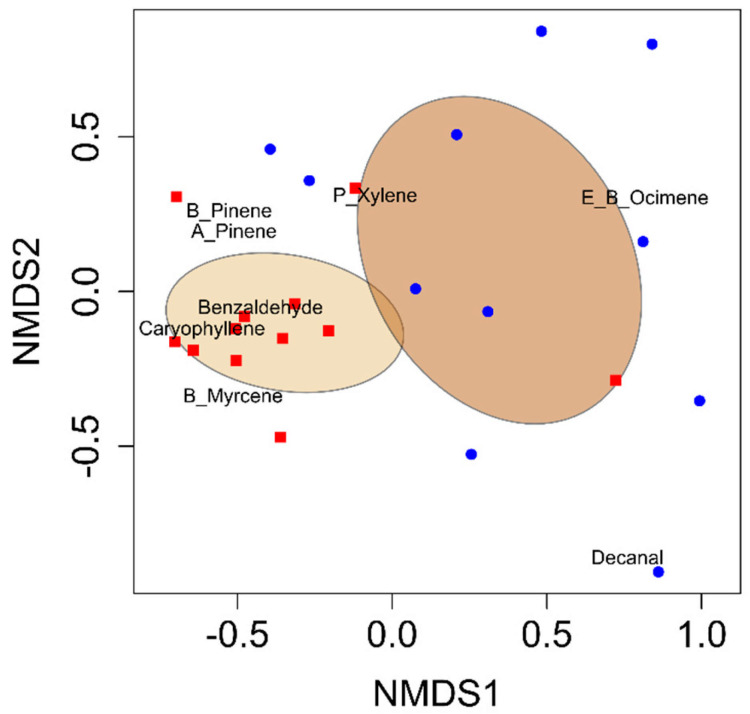
Non-metric multidimensional scaling (NMDS) ordination plot showing VOC blends from developing cotton bolls (blue) compared to cotton bolls damaged by adult leaffooted bug herbivory (red). Ellipses describe the variation of samples around the treatment centroid and represent 95% confidence intervals.

Eight volatile compounds were identified in the headspace samples of developing cotton bolls across both herbivory and control treatments. The observed differences were quantitative, indicating differences in emission levels rather than qualitative differences in the presence or absence of specific compounds. All eight compounds were consistently detected in both treatments. These compounds were (*E*)-β-ocimene, α-pinene, β-pinene, β-myrcene, (*E*)-β-caryophyllene, p-xylene, benzaldehyde, and decanal (Table 1). The VOC blend emitted by developing cotton bolls damaged by adult leaffooted bugs differed significantly from that of non-damaged control bolls (PERMANOVA: *F*_1,22_ = 6.32, *p* = 0.001; Figure 1). Random Forest analysis identified benzaldehyde and α-pinene as the primary compounds distinguishing the VOC blends between developing cotton bolls damaged by adult leaffooted bugs and non-damaged (control) developing cotton bolls (Figure S1). Total VOC emissions from damaged bolls were significantly greater than those from non-damaged bolls (*t*_22_ = 4.71, *p* = 0.0001) (Table 1). Six compounds showed significant increases in response to adult leaffooted bug herbivory: benzaldehyde (*t*_22_ = 4.52, *p* = 0.0002), α-pinene (*t*_22_ = 2.53, *p* = 0.009), β-pinene (*t*_22_ = 2.28, *p* =0.02), β-myrcene (*t*_22_ = 2.52, *p* = 0.009), (*E*)-β-caryophyllene (*t*_22_ = 3.66, *p* = 0.001), and p-xylene (*t*_22_ = 1.71, *p* = 0.05) (Table 1). Moreover, although some volatile compounds were detected in the quality control collections, these were not plant-derived and were excluded from further analysis. Therefore, only VOCs associated with cotton bolls in response to leaffooted bug herbivory were preserved in the final results.

### 3.2. Leaffooted Bugs Are Attracted to HIPVs from Cotton Bolls

**Figure 2:**
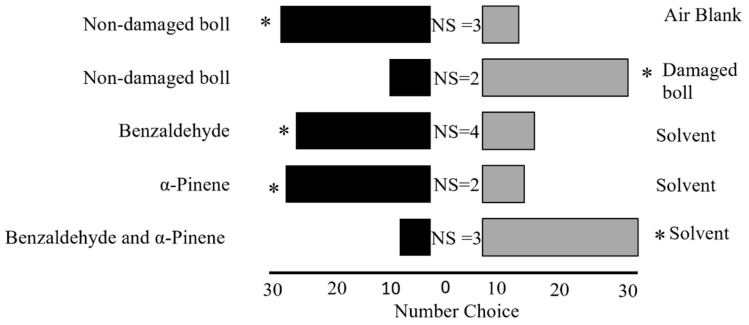
Behavioral preference of the adult leaffooted bug in a Y-tube olfactometer bioassay. For each pair of treatments, 36 adult leaffooted bugs were tested. NS: non-responsive. Asterisks indicate a statistically significant preference; all comparisons were significant (*p* < 0.05).

Our results showed that adult leaffooted bugs exhibited a strong preference for non-damaged (control) developing cotton bolls over air blanks (Goodness-of-fit test, *n* = 36, χ^2^ = 13.4, *df* = 1, *p* = 0.0002; Figure 2). We also observed that adult leaffooted bugs showed a preference for developing cotton bolls damaged by adult leaffooted bugs compared to the non-damaged (control) developing cotton bolls (*n* = 36, χ^2^ = 11.1, *df* = 1, *p* = 0.0009; Figure 2). Building on this finding, we evaluated the preference of adult leaffooted bugs for individual synthetic HIPVs in comparison to solvent control. We found that adult leaffooted bugs exhibited a significant preference for benzaldehyde compared to the control solvent (dichloromethane) (*n* = 36, χ^2^ = 7.1, *df* = 1, *p* = 0.008; Figure 2). Similarly, they demonstrated a strong preference for α-pinene over the control solvent (dichloromethane) (*n* = 36, χ^2^ = 11.1, *df* = 1, *p* = 0.0009; Figure 2). Interestingly, when these two compounds were presented in combination, adult leaffooted bugs preferred the control solvent (dichloromethane) over the blend containing benzaldehyde and α-pinene (*n* = 36, χ^2^ = 16,0 *df* = 1, *p* = 0.0006; Figure 2).

## 4. Discussion

Behavioral responses revealed that adult leaffooted bugs were attracted to cotton bolls. Moreover, they show a preference for HIPVs from bolls damaged by conspecific adult leaffooted bugs over VOCs from non-damaged bolls. Specific compounds in the HIPV blend emitted by damaged bolls were analyzed, showing that adult leaffooted bugs were attracted to benzaldehyde and α-pinene individually but repelled when the two compounds were combined. This study builds on previous research examining the induction of HIPVs in cotton caused by herbivory from hemipteran pests [17,22,32,33,34,35]. These findings emphasize the significant role of HIPVs in influencing pest behavior. A better understanding of the natural mechanisms of HIPV induction in cotton could support the development of sustainable pest control methods to manipulate leaffooted bug behavior.

While many previous studies on volatile emissions from cotton in response to piercing–sucking insect damage focused on emissions from leaves [32,33,36], our findings highlight the distinctive effects of such herbivory on cotton bolls. This study revealed that adult leaffooted bug herbivory significantly altered VOC emissions from cotton bolls, increasing the concentrations of compounds such as benzaldehyde, α-pinene, β-pinene, β-myrcene, (*E*)-β-caryophyllene, and p-xylene. A prior laboratory study similarly found that hemipteran herbivory, including *L. phyllopus*, caused a 1.8-fold increase in volatile emissions and significantly higher levels of terpenes compared to non-damaged bolls [22]. Previous studies with other families of hemipteran pests on cotton also aligned with our results. Significantly higher emissions were observed from bolls exposed to the southern green stink bug, *N. viridula* (Linnaeus), and the brown stink bug, *E. servus* (Say), compared to controls. Additionally, herbivory by *E. servus* and *N. viridula* significantly increased the emission of several terpenes, including β-ocimene and β-farnesene, compared to the controls [17]. The consistency of our results with previous studies highlights the role of hemipteran herbivory in altering cotton plant volatile blends. The increased release of volatiles in response to adult leaffooted bug herbivory provides valuable ideas into plant–insect interactions and their ecological implications, offering practical solutions for effective pest control.

The bioassay study found also that adult leaffooted bugs prefer HIPVs released by bolls damaged by conspecifics over VOCs from non-damaged bolls. Similarly, *Thrips tabaci* (Lindeman) (Thysanoptera: Thripidae) exhibited a preference for cotton seedlings with foliar damage caused by conspecifics or *Tetranychus urticae* (Koch) (Acari: Tetranychidae), its prey, over undamaged cotton seedlings [37]. In contrast to our findings, previous studies suggest that many herbivorous insects use HIPVs to avoid plants already occupied by herbivores [38]. This avoidance behavior likely benefits herbivores by reducing their offspring’s exposure to predation, competition from older insects, and the performance costs associated with induced plant defenses [39]. For example, a previous study reported that various cotton HIPVs repel the bug *Apolygus lucorum* (Meyer-Dür) (Hemiptera: Miridae) [40]. These differences in herbivore behavior highlight the importance of characterizing behavioral responses for specific plant–insect species combinations. Although our bioassay showed valuable results, a few factors may influence the results and interpretation of behavioral assays conducted under controlled laboratory conditions. For instance, the laboratory setting of the olfactometer may not fully replicate the complexity of natural environments in which leaffooted bugs exhibit their typical behaviors, potentially influencing their responses to VOCs. Moreover, the dual-choice design of the Y-tube olfactometer limits leaffooted bugs to only two options, which may not accurately represent the broader range of cues and choices available under field conditions.

Interestingly, our findings showed that adult leaffooted bugs were repelled by the combination of benzaldehyde and α-pinene, despite being attracted to each compound individually. Behavior studies have shown that exposing herbivores to specific blends or combinations of VOCs often produced stronger behavioral responses than single VOCs alone [11]. Generally, when VOCs are identified individually rather than as part of a specific blend, they may be interpreted as cues from non-host plants. In contrast, when these VOCs are combined, they can convey a completely altered cue [41]. Herbivores may recognize host plants either through species-specific VOCs or by detecting particular ratios of common VOCs [42]. A previous study aligned with our results showed that the cue compounds cis-jasmone and cis-3-hexenyl acetate, at specific doses, enhanced the parasitic efficiency of *Campoletis chlorideae* (Uchida) (Hymenoptera: Ichneumonidae), an important larval endoparasitoid of the cotton bollworm *Helicoverpa armigera* (Hübner). However, when these compounds were combined, the mixture canceled this beneficial effect [43].

In the current study, several potential explanations could account for the observed repulsion of leaffooted bugs in relation to the combination of benzaldehyde and α-pinene. The attractiveness of benzaldehyde and α-pinene individually may have been strongly influenced by their concentrations, as changes in their concentrations within a blend can result in different, or even opposing, behavioral responses. Additionally, it is possible that the concentration ratios used in the synthetic blend did not accurately reflect the natural ratios emitted by damaged bolls, potentially causing a shift from attraction to repellence. Moreover, the combination of benzaldehyde and α-pinene may mask certain cues that leaffooted bugs rely on for host location. When the blend is highly concentrated or differs from its natural ratio, it may no longer be recognized as a favorable cue, potentially resulting in avoidance behavior. Other studies have suggested that intercropped plants can mask or disrupt the olfactory cues herbivores rely on to locate their host plants, thereby reducing herbivore attraction and feeding [44].

As an eco-friendly alternative to conventional pesticides, VOCs offer a sustainable approach to managing herbivorous pests. Moreover, the global effectiveness and availability of traditional pesticides are declining due to the evolution of pest resistance and increasingly strict regulatory restrictions, highlighting the crucial need for alternative pest management approaches [45]. Although VOCs offer sustainable advantages for environmentally friendly pest management, their practical implementation in the field remains challenging. It is difficult to replicate the precise concentrations used in laboratory settings in the field, which can affect the effectiveness of herbivores responses under real-world conditions [46]. VOCs can degrade quickly when exposed to environmental oxidants such as NOx or ozone, which significantly reduces their persistence and effectiveness in the field [47]. Furthermore, developing effective synthetic VOC formulations is both costly and time-consuming, requiring extensive screening and optimization [46]. For these compounds to be broadly adopted in pest management, key challenges related to their stability, field applicability, and cost-effectiveness must be solved.

Identifying volatile blends emitted from cotton bolls in response to leaffooted bug feeding helps to clarify their role in mediating cotton plant–leaffooted bug interactions. These HIPVs influence leaffooted bug host selection and aggregation behavior. Understanding these interactions is important for developing environmentally sustainable pest management strategies and reducing reliance on broad-spectrum insecticides. Our results indicate that leaffooted bugs are attracted to benzaldehyde and α-pinene, which could be used to develop attractant-based traps for early leaffooted bug detection and monitoring in cotton fields. Moreover, these HIPVs could also be incorporated into attract-and-kill strategies by combining them with insecticides. While our study provides valuable information on HIPV emissions in response to leaffooted bug herbivory in cotton cultivation, we acknowledge a few limitations that may have influenced our findings. One limitation of this study is that we did not conduct a field trial. Implementing a field study would be a valuable next step to assess leaffooted bug attraction to VOCs under natural conditions. Another limitation of this study is that only two VOCs, benzaldehyde and α-pinene, were tested, and their combination produced an effect opposite to that observed when the compounds were tested individually. In addition, it is possible that other VOCs, either those identified in the Random Forest analysis with lower mean decrease Gini scores or those not included in the Y-tube olfactometer bioassay due to the study’s specific objectives, may also influence leaffooted bug behavior. Furthermore, we evaluated only a single concentration. It is possible that different behavioral results might occur at other concentrations, suggesting that concentration-dependent effects could play a significant role in influencing leaffooted bug responses.

Future research could compare the responses of males, females, and juveniles, as this study did not report the sex of the leaffooted bugs and included only a single age class. Males and females may respond differently to the same VOCs due to physiological or behavioral differences. Understanding sex-specific responses could aid in the development of more targeted and effective pest management strategies. Additionally, future field studies should evaluate the effectiveness of benzaldehyde and α-pinene, alone or in combination with adult leaffooted bug aggregation pheromones, in baited field traps. Additionally, future research should investigate potential differences in the quality and quantity of VOCs emitted by different cotton cultivars, as these variations may be associated with their attractiveness to leaffooted bugs or their potential utility in pest management. For example, certain cultivars may emit higher levels of specific attractant or repellent VOCs, thereby influencing the chances of leaffooted bug infestation.

## Figures and Tables

**Table 1:** Volatile emissions (mean (ng) ± SD) from either non-damaged boll (control) or damaged boll by adult leaffooted bug were collected after five hours of sampling.

Volatile Compound	Damaged Boll	Non-Damaged Boll	*t*-Test	*p*-Values
benzaldehyde *	36.8 ± 17.4	12.2 ± 7.2	4.52	0.0002
α-pinene *	13.2 ± 11.2	4.3 ± 5.0	2.53	0.009
β-myrcene *	3.8 ± 4.4	0.53 ± 1.0	2.52	0.009
β-pinene *	2.1 ± 1.6	0.8 ± 1.2	2.28	0.02
(*E*)-β-caryophyllene *	2.5 ± 2.0	0.4 ± 0.7	3.66	0.001
P-xylene *	4.2 ± 3.1	2.1 ± 3.0	1.7	0.05
decanal	4.6 ± 3.7	8.0 ± 10.2	1.11	0.28
(*E*)-β-ocimene	1.7 ± 1.4	3.6 ± 6.2	1.01	0.32
Total *	68.9 ± 24.0	31.8 ± 12.8	4.71	0.0001

An asterisk (*) indicates compounds with significantly increased emissions because of adult leaffooted bug herbivory (exact *p* values given). Student’s *t*-test was used to compare the emissions of VOCs from individual compounds and total compounds between damaged bolls and non-damaged bolls. *n* = 24. Degrees of freedom (DF) = 22.

## Data Availability

The original contributions presented in this study are included in the article/Supplementary Material. Further inquiries can be directed to the corresponding author.

## Supplementary Materials

The following supporting information can be downloaded at: https://www.mdpi.com/article/10.3390/insects16040425/s1, Figure S1: Random forest analyses for the VOC blends from developing cotton bolls with adult leaffooted bug herbivory and non-damaged (control) developing bolls. Gini scores indicate the contributions of each compound to distinguish between treatments.
