# Supplementary material for: Leaffooted Bugs, Leptoglossus phyllopus (Hemiptera: Coreidae), Are Attracted to Volatile Emissions from Herbivore-Damaged Cotton Bolls
Source: Insects. 2025 Apr 17;16(4):425. doi: 10.3390/insects16040425 (PMC12027685; doi:10.3390/insects16040425)
Supplement: Supplementary file 1 [file insects-16-00425-s001.zip › insects-3518167-supplementary.pdf]

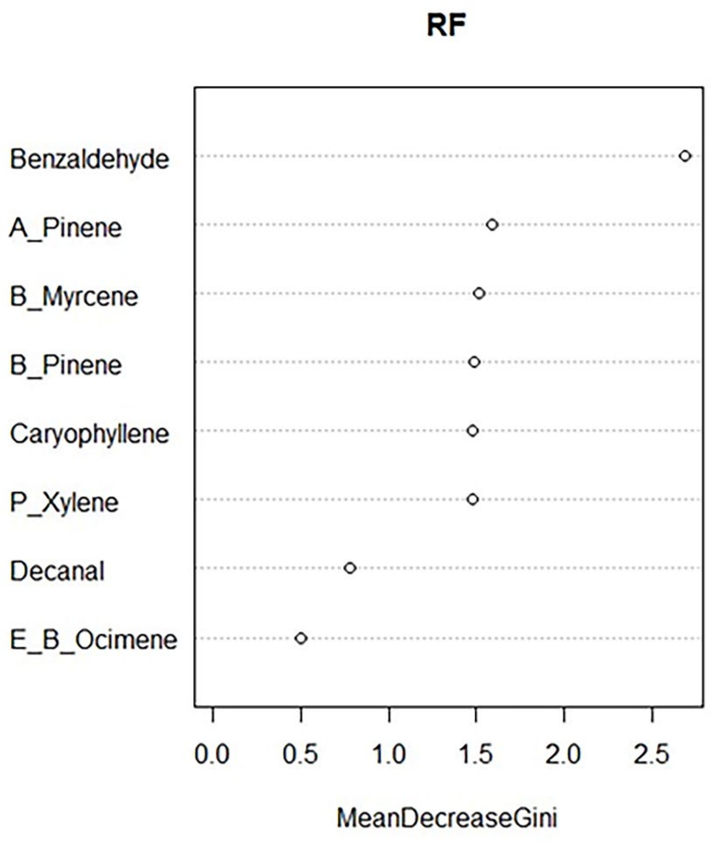

Figure S1. Random forest analyses for the VOC blends from developing cotton bolls with adult leaffooted bug herbivory and non-damaged (control) developing bolls. Gini scores indicate the contributions of each compound to distinguish between treatments
